# Impact of Imatinib Intake Timing on Oncological Outcomes in Metastatic Gastrointestinal Stromal Tumors (GISTs): A Retrospective Single-Institute Study

**DOI:** 10.7759/cureus.109890

**Published:** 2026-05-29

**Authors:** Kenza Bahida, Mohamed El Fadli, Othmane Zouiten, Leila Afani, Rhizlane Belbaraka

**Affiliations:** 1 Medical Oncology, Mohammed VI University Hospital, Marrakech, MAR; 2 Biosciences and Health Laboratory, Faculty of Medicine and Pharmacy, Cadi Ayyad University, Marrakech, MAR

**Keywords:** chronology, chrono pharmacology, gastrointestinal stromal tumor (gist), gist management, imatinib therapy, oncological general surgery

## Abstract

Background: The time-of-day administration significantly influences treatment outcomes in various cancer settings, particularly with cytotoxic chemotherapy and immunotherapy. However, data regarding tyrosine kinase inhibitors are scarce. We aimed to investigate whether the circadian timing (morning versus evening) of imatinib administration impacts progression-free survival (PFS) in patients with metastatic gastrointestinal stromal tumors (GISTs). A secondary objective was to evaluate the impact of clinical and pathological factors, such as primary tumor site and metastatic burden, on oncological outcomes.

Methods: We conducted a single-center retrospective study on patients with histologically confirmed metastatic GISTs, treated with first-line imatinib (400 mg/day). Patients were categorized into "Morning" (before 12 PM) or "Evening" (after 12 PM) intake groups. The primary endpoint was PFS. Survival curves were estimated using the Kaplan-Meier method and compared using the log-rank test.

Results: Forty-four patients were included (median age 54.5 years). The primary tumor sites were mainly gastric n= 20 (45%) and small intestine n= 16 (36%). Hepatic metastases were present in 82% of cases (n= 36). Imatinib was administered in the morning for 59% of patients (n=26) and in the evening for 41% (n=18). Median PFS was 61.5 months in the Morning group versus 26.2 months in the Evening group. Despite this numerical difference, the analysis revealed no statistically significant difference (p=0.22). Gastric primary site (p=0.017) and low metastatic burden (p<0.01) were confirmed as significant favorable prognostic factors.

Conclusion: In this cohort, no statistically significant difference in oncological outcomes was detected based on the circadian timing of Imatinib administration. While this suggests a flexible dosing schedule tailored to patient tolerance is reasonable in routine practice, larger, appropriately powered studies incorporating mutational profiling are required to definitively rule out a time-dependent therapeutic effect.

## Introduction

Gastrointestinal stromal tumors (GISTs) represent the most common mesenchymal neoplasms of the digestive tract. The management of metastatic or unresectable disease was revolutionized by the introduction of imatinib mesylate, a tyrosine kinase inhibitor (TKI) targeting KIT and PDGFRA receptors [1]. While the standard dosage of 400 mg daily is well-established, optimizing the administration schedule remains a subject of clinical interest to improve adherence and minimize toxicity.

Time-of-day administration, which adapts drug delivery to the body's circadian rhythms, has proven relevant in oncology. Seminal works have demonstrated that the efficacy and toxicity of cytotoxic agents, such as fluoropyrimidines and platinum salts, vary significantly depending on the time of intake due to circadian fluctuations in hepatic metabolism and cellular proliferation [2,3]. More recently, evidence has emerged suggesting that immune checkpoint inhibitors (ICIs) may also exhibit time-dependent efficacy, likely modulated by the circadian nature of the immune system [4].

However, the application of time-specific administration schedules to oral targeted therapies remains an underexplored frontier. While imatinib possesses a relatively long half-life (approximately 18 hours), its pharmacokinetics are subject to significant inter-individual variability driven by CYP3A4 and CYP2C8 activity [5], both of which exhibit circadian oscillations. Furthermore, data from related TKIs, such as sunitinib, have demonstrated that administration timing can significantly alter trough plasma concentrations (Ctrough​), with morning intake potentially resulting in lower drug exposure compared to evening dosing. Given that achieving a threshold steady-state concentration (Cmin​>1100ng/mL) is a known predictor of clinical benefit in GISTs [6], the circadian timing of intake may be a critical, yet overlooked, determinant of therapeutic success.

In clinical practice, the choice between morning and evening intake remains largely empirical. Therefore, we aimed to investigate whether the circadian timing (morning versus evening) of imatinib administration impacts progression-free survival (PFS) in patients with metastatic GISTs treated in a real-world setting. A secondary objective was to evaluate the impact of baseline clinical and pathological factors, such as primary tumor site and metastatic burden, on oncological outcomes.

## Materials and methods

Study design and population

We conducted a retrospective, single-center longitudinal study to evaluate the impact of time-of-day imatinib administration on oncological outcomes. The study population consisted of patients diagnosed with metastatic or unresectable GISTs who were managed at the Medical Oncology Department of Mohammed VI University Hospital.

Given the rare incidence of GISTs and the stringent inclusion criteria requiring detailed documentation of medication timing, a formal a priori sample size calculation was not performed. Instead, a convenience sample comprising all eligible consecutive patients treated at our institution who met the strict data requirements was utilized. Furthermore, due to the retrospective nature of the study and the evolving landscape of molecular diagnostics during the early inclusion period, genotyping for KIT and PDGFRA mutations was not systematically performed for all patients. Due to this limited availability of routine molecular testing, mutational status was not utilized as a stratification factor in the survival analysis, reflecting the pragmatic, real-world constraints of the cohort.

Inclusion and exclusion criteria

To ensure a homogenous study population and minimize confounding variables, strict eligibility criteria were applied. Patients were required to have a histologically confirmed diagnosis of GISTs, regardless of the primary site of origin, and present with metastatic or unresectable disease at the time of imatinib initiation.

Additionally, eligible individuals must have received imatinib mesylate as their first-line systemic therapy. Patients were required to have documented adherence and consistent intake of the drug either in the morning or evening. Because pharmacy refill records were not systematically available, adherence and administration timing were extracted from a retrospective review of physician consultation notes and nursing records. To be categorized, patients must have maintained their designated intake schedule (morning or evening) for at least 80% of their treatment duration.

Conversely, patients were excluded if they were treated exclusively in the adjuvant setting for localized disease or had received prior TKIs or other systemic therapies before initiating imatinib. Furthermore, cases were excluded if they presented with incomplete medical records, specifically lacking adequate details regarding follow-up duration, radiological response, or the precise timing of medication administration. Patients who permanently shifted their intake time between morning and evening due to gastrointestinal toxicity or patient preference were also excluded to prevent exposure misclassification.

Treatment protocol and intake timing definition

All patients were started on a standard initial dose of 400 mg/day of imatinib mesylate, administered orally. In instances of documented radiological disease progression, dose escalation to 800 mg/day was permitted according to institutional protocols and clinician discretion.

For the purpose of this chronobiological analysis, the study population was bifurcated based on the self-reported and medically recorded timing of drug administration, Morning Group, defined as the consistent intake of imatinib before 12:00 PM. Evening Group, defined as the consistent intake of imatinib after 12:00 PM.

Data collection

Using a standardized data collection form, we extracted comprehensive clinical and pathological data from institutional electronic medical records and physical charts. The baseline variables collected encompassed patient demographics, specifically age at diagnosis and sex, as well as detailed tumor characteristics. These included the primary tumor site (stomach, small intestine, or other), metastatic burden (quantified by the number of lesions), and specific metastatic locations (e.g., liver, peritoneum). 

While initial data collection efforts attempted to capture required dose modifications and documented side effects, the retrospective nature of the study yielded heavily fragmented and incomplete records regarding exact dose escalation timelines and low-grade toxicities. Consequently, variables related to adverse events and dose escalations were excluded from the final quantitative analysis to avoid reporting bias.

Statistical analysis

The primary endpoint of the study was PFS, defined as the interval from the date of imatinib initiation to the date of first documented radiological progression (per RECIST 1.1 criteria) or death from any cause. Patients alive and without progression at the time of the last follow-up were censored.

Descriptive statistics were used to summarize baseline characteristics, with categorical variables expressed as frequencies and percentages, and continuous variables as means (±SD) or medians (IQR). Survival curves were estimated using the Kaplan-Meier method and compared using the two-sided log-rank test. A p-value of < 0.05 was established as the threshold for statistical significance.

All computational analyses were performed using Python (version 3.8.10). Survival analysis was conducted using the lifelines (v0.27.0) library, while data manipulation and descriptive statistics were handled via the pandas (v1.3.5) and numpy (v1.21.0) packages.

## Results

Patient characteristics

**Figure 1:**
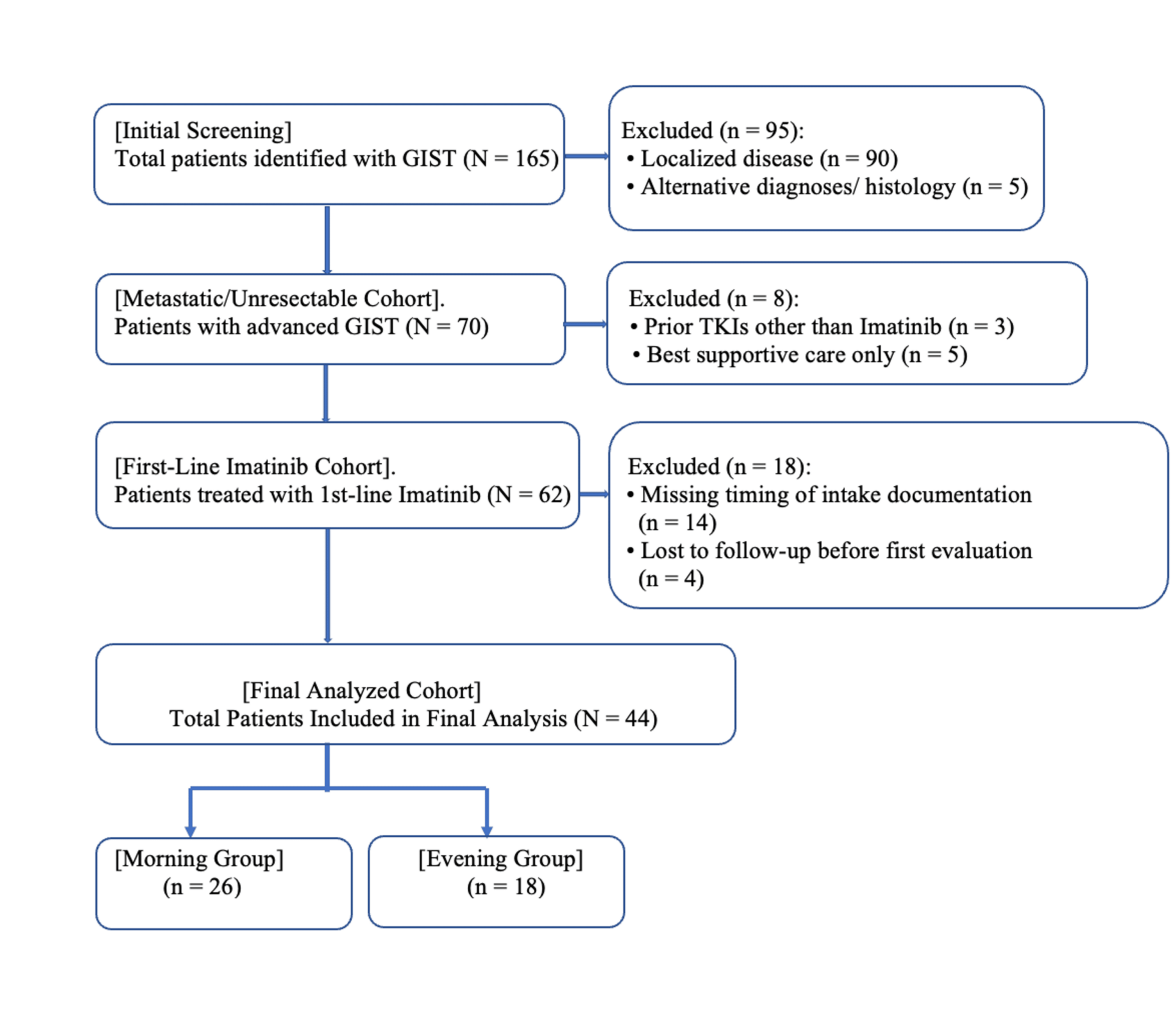
Flow chart of patient selection. N: number of patients; GIST: gastrointestinal stromal tumor; TKI: tyrosine kinase inhibitor.

The patient selection process is detailed in the study flow chart (Figure 1). An initial search of the institutional database identified 165 patients with a diagnosis of GISTs during the study period.

After excluding 95 patients (57.6% of the initial screening), primarily those with localized disease treated in the adjuvant setting, and eight patients (4.8%) who received best supportive care or alternative first-line TKIs, a cohort of 62 patients (37.6%) treated with first-line imatinib for metastatic or unresectable disease was identified.

A further 18 patients (10.9% of the total) were excluded due to loss to follow-up or incomplete medical records, specifically lacking consistent documentation of the circadian timing of drug intake. The final cohort selected for statistical analysis comprised 44 patients (26.7% of the original screened population), categorized into the Morning intake group (n = 26; 59.1%) and the Evening intake group (n = 18; 40.9%).

A total of 44 patients were included in the final analysis. The median age of the cohort was 54.5 years, with a range of 38 to 81 years. The study population exhibited an even gender distribution, with a male-to-female ratio of 1:1.

Regarding the primary tumor origin, the stomach was the most frequent site of disease, accounting for 45% of cases (n=20). This was followed by the small intestine in 36% of patients (n=16) and the rectum in 18% (n=8).

In terms of metastatic dissemination, the majority of patients (n=30), 68% presented with a single metastatic site. The liver was the predominant organ involved, identified as the site of metastasis in 36 patients (82%).

Impact of imatinib intake timing (primary endpoint)

Regarding the circadian timing of treatment, imatinib was administered in the morning for the majority of the cohort (n=26), 59%, while the remaining 18 patients (41%) took the medication in the evening (Table 1).

**Table 1:** Baseline demographic and clinical characteristics of the study population (N=44).

Characteristic	Number of Patients (n)	Percentage (%)
Total Population	44	100%
Age (years)		
Median	54.5	-
Range	38 – 81	-
Gender		
Male	22	50.00%
Female	22	50.00%
Primary Tumor Site		
Stomach	20	45.40%
Small Intestine	16	36.40%
Rectum	8	18.20%
Metastatic Burden		
Single Site	30	68.20%
Multiple Sites	14	31.80%
Dominant Metastatic Site		
Liver	36	81.80%
Lung	6	13.60%
peritoneum	2	4,6%
Imatinib Intake Timing		
Morning	26	59.10%
Evening	18	40.90%

**Figure 2:**
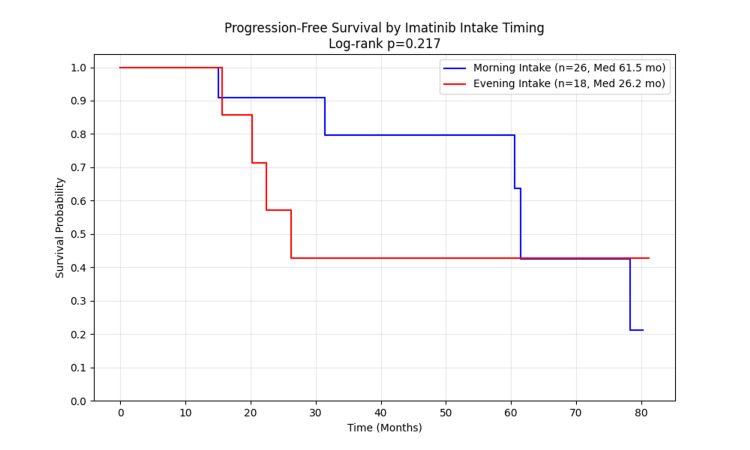
Kaplan-Meier estimates of progression-free survival according to imatinib intake timing. Blue curve: Morning Intake (n=26, Median PFS 61.5 months). Red curve: Evening Intake (n=18, median PFS 26.2 months). Log-rank p-value = 0.22 (non-significant). PFS: progression-free survival

The analysis of the primary endpoint revealed a median PFS of 61.5 months for the morning intake group compared to 26.2 months for the evening intake group. Despite the numerical difference favoring the morning schedule, the log-rank analysis demonstrated no statistically significant difference between the two groups (p=0.22). The Kaplan-Meier survival curves (Figure 2) illustrate this lack of significant separation.

Analysis of other prognostic factors

**Figure 3:**
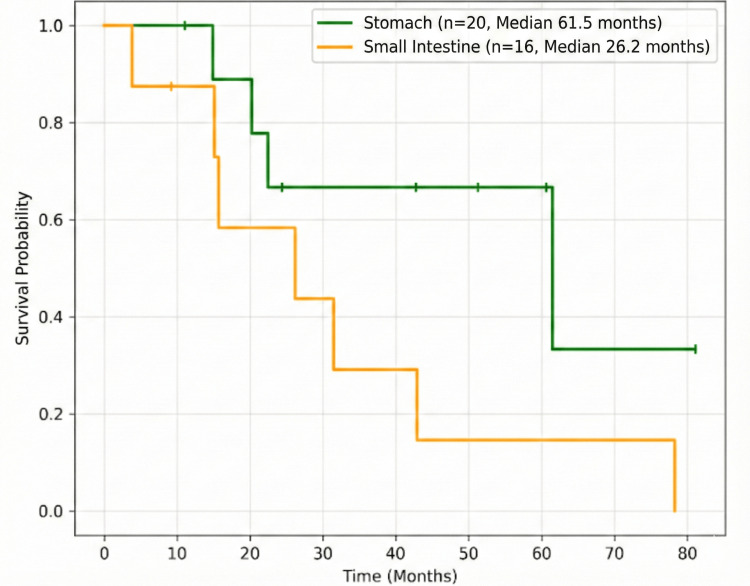
Kaplan-Meier estimates of PFS according to the primary tumor site. Gastric GISTs (green) demonstrate superior survival compared to small intestine GISTs (orange). Log-rank p-value = 0.017. PFS: progression-free survival; GISTs: gastrointestinal stromal tumors

Univariate analysis was performed to validate established prognostic markers within our cohort. The primary tumor location significantly influenced outcomes; patients with gastric GISTs demonstrated superior survival compared to those with intestinal GISTs (p=0.017) (Figure 3).

**Figure 4:**
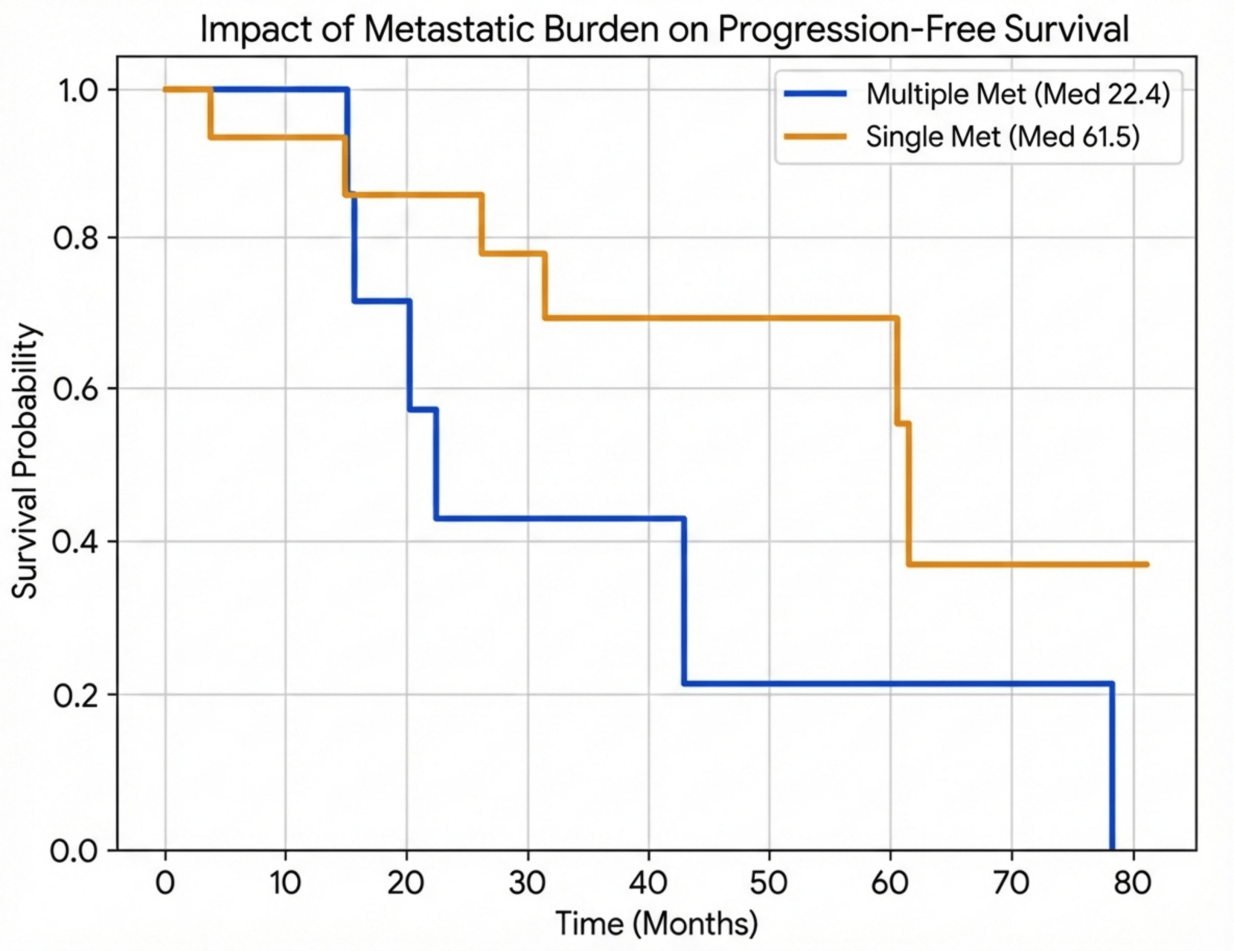
Impact of metastatic burden on progression-free survival. Survival analysis comparing patients with a single metastatic site versus those with multiple metastatic sites (≥2). Patients with a low tumor burden (single site) exhibit significantly prolonged survival compared to those with multiple metastases. The difference is statistically significant (p<0.01). Single Met: single metastasis; multiple Met: multiple metastases.

Furthermore, the extent of metastatic burden was confirmed as a strong predictor of survival. Patients presenting with a solitary metastatic site exhibited significantly longer PFS compared to those diagnosed with multiple metastatic sites (p<0.01) (Figure 4).

## Discussion

This study explored whether the circadian timing of imatinib administration (morning vs evening) influences PFS in patients with metastatic GISTs. Although a difference in median PFS was observed (61.5 months for morning vs. 26.2 months for evening), this was not statistically significant (p = 0.22). Tumor location, type of metastases, and number of metastatic sites, however, were significantly associated with survival in our univariate analysis, aligning with established prognostic factors in GISTs.

While the impact of imatinib timing was not statistically significant in our study, the trend observed and the emerging literature on time-of-day administration suggest that treatment timing could influence therapeutic outcomes. 

From a mechanistic perspective, time-specific scheduling is supported by data showing that drug absorption, metabolism, distribution, and cellular sensitivity fluctuate throughout the day due to circadian control of enzymes, transporters, and cell cycle checkpoints [7,8]. For example, CYP3A4, a key enzyme involved in imatinib metabolism, exhibits circadian variation in activity [9], which could influence drug levels and therapeutic outcomes depending on the timing of administration.

The alignment of treatment timing with biological rhythms has been explored in various cancers and treatment regimens. For example, in metastatic colorectal cancer, Lévi et al. showed improved efficacy and reduced toxicity when chemotherapy was administered according to circadian rhythms [10]. This concept has been reinforced in other studies where irinotecan, a topoisomerase I inhibitor, demonstrated sex-specific time-dependent toxicity; men tolerated morning administration better, while women benefited from afternoon dosing [11]. 

Similarly, in glioblastoma, a better overall survival was demonstrated with morning administration of temozolomide in patients with MGMT-methylated tumors, possibly due to time-dependent expression of deoxyribonucleic acid (DNA) repair genes [12].

Furthermore, in ovarian cancer, circadian-timed chemotherapy using doxorubicin in the morning and cisplatin in the evening led to fewer adverse effects, particularly nephrotoxicity, suggesting a protective role of circadian timing on organ-specific toxicity [13]. In pediatric leukemia, one retrospective analysis revealed that evening chemotherapy led to a nearly twofold improvement in five-year disease-free survival, again highlighting the potential survival benefits of timing therapy [14].

Similarly, in glioblastoma, a better overall survival was demonstrated with morning administration of temozolomide in patients with MGMT-methylated tumors, possibly due to time-dependent expression of DNA repair genes [12].

In addition to these studies, recent attention has turned toward the time-dependent pharmacokinetics of tyrosine kinase inhibitors like sunitinib, a multikinase inhibitor used in renal cell carcinoma and GISTs. Preclinical studies have shown that sunitinib and its active metabolite SU12662 exhibit circadian fluctuations in plasma levels, with significantly higher exposure when administered at 4 a.m. and 4 p.m. compared to 8 a.m. or 8 p.m. A clinical pharmacokinetic study further confirmed that trough concentrations were higher when sunitinib was taken in the afternoon (1 p.m. or 6 p.m.) versus in the morning (8 a.m.), suggesting that administration time may influence systemic drug exposure [6].

However, a prospective trial comparing morning versus evening administration of sunitinib in patients with renal cell carcinoma did not find significant differences in toxicity, efficacy, or quality of life [15]. Despite the neutral results, the study was limited by a small sample size and high dropout rate, underscoring the challenges of studying treatment timing in clinical settings.

More recently, the timing of ICIs has garnered attention. Recent meta-analyses and observational studies in melanoma and lung cancer suggest that morning administration of programmed cell death protein 1/ anti-programmed death-ligand 1 inhibitor (PD-1/PD-L1 inhibitor) agents is associated with better overall survival compared to late afternoon administration [16]. This is likely driven by the circadian clock of the immune system, where antigen presentation and T-cell priming are more efficient in the early active phase of the day.

Despite these promising precedents, our findings did not demonstrate a statistically significant impact of intake timing in the context of imatinib. Several factors may explain this. First, the retrospective design and modest sample size severely limit statistical power; consequently, our failure to detect a statistically significant difference (p = 0.22) cannot be interpreted as definitive proof of therapeutic equivalence or that efficacy is entirely independent of intake timing. Second, routine genotyping for KIT and PDGFRA mutations was not systematically available for this cohort. Because mutational status is a profound driver of imatinib response in GISTs, its absence as a stratification factor represents a major unmeasured confounder that could potentially mask true survival differences between the intake groups. Third, imatinib has a long half-life (~18 hours), which could attenuate circadian fluctuations in plasma concentration compared to therapies with shorter half-lives. Finally, patient adherence and precise timing of intake were extracted from medical records and may not fully capture real-world variations or minor deviations in daily schedules.

Nevertheless, the difference in median PFS observed between morning and evening administration warrants further investigation, particularly in light of the consistent survival trend favoring morning dosing. Prospective trials with pharmacokinetic monitoring and circadian gene expression profiling may help clarify whether specific patient subgroups could benefit from tailored administration timing.

Our findings contribute novel data to the underinvestigated area of time-of-day imatinib administration in GIST management. While this retrospective cohort did not demonstrate a statistically significant survival benefit based on intake timing, the study was underpowered to definitively establish therapeutic equivalence. The integration of circadian principles into future, larger-scale, molecularly stratified trials remains a promising strategy to optimize targeted therapies.

## Conclusions

In this small retrospective cohort, despite a numerically large difference in median PFS favoring morning administration (61.5 vs. 26.2 months), the timing of imatinib intake did not reach statistical significance as an independent prognostic factor.

Due to the limited sample size and the retrospective nature of the analysis, further adequately powered, prospective studies are required to determine if circadian timing influences imatinib efficacy.
